# Impact of injury-related mortality on life expectancy in Zhejiang, China based on death and population surveillance data

**DOI:** 10.1186/s12889-017-4566-3

**Published:** 2017-07-17

**Authors:** Fang-Rong Fei, Jie-Ming Zhong, Min Yu, Wei-Wei Gong, Meng Wang, Jin Pan, Hai-bin Wu, Ru-Ying Hu

**Affiliations:** Department of NCDs Control and Prevention, Zhejiang Provincial Centre for Disease Control and Prevention, 3399 Binsheng Road, Hangzhou, 310051 China

**Keywords:** Injury-related deaths, Life expectancy, Loss of life

## Abstract

**Background:**

Life expectancy is a statistical measure of the average time an organism is expected to live. The purpose of this study was to evaluate the impact of injury-related mortality on life expectancy in Zhejiang Province.

**Methods:**

Our study used standard life tables to calculate life expectancy and cause-removed life expectancy based on mortality data from the Zhejiang Chronic Disease Surveillance System.

**Results:**

Life expectancy of residents in Zhejiang was 77.83 years in 2013, with females having a higher life expectancy than males. The decrease in life expectancy caused by injury-related deaths was 1.19 years, the effect of which was reduced for females and urban residents compared with males and rural residents. The greatest impact on life expectancy was road traffic injuries (RTIs), (0.29 years lost overall, 0.36 for men vs. 0.21 for women and 0.26 for urban residents vs. 0.31 for rural residents). The main causes were falls (0.29 years lost overall, 0.30 for men vs. 0.28 for women and 0.28 for urban residents vs. 0.30 for rural residents), followed by drowning (0.15 years lost), suicide (0.11 years lost), and poisoning (0.04 years). For children less than 5 years old and elders aged over 65, drowning had a greater impact than falls.

**Conclusions:**

Our findings indicate that injury deaths had a major impact on life expectancy in Zhejiang. More attention should be paid to road traffic injury, and preventive action should be taken to reduce injury-related deaths to increase life expectancy, especially in children under five years of age and the elders over 65 years of age.

## Background

Life expectancy is a statistical measure of the average time an organism is expected to live based on current mortality [1]. It is a significant indicator for assessing the health and economic and social development levels of residents [2–4]. Zhejiang Province is located along the shore of the East China Sea, and covers an area of 101.8 thousand square kilometres. It is comprised of 11 cities and 90 districts. The total population of the province is 55.08 million. Life expectancy increased from 74.88 years to 77.57 years between 2000 and 2012 in Zhejiang Province. However, more than 5 million residents die from injury each year, which accounts for 9.8% of global deaths [5]. The proportion of injury-related deaths increased from 18.69% in 2004 to 21.26% in 2011 among those aged 0–14 years [6]. Injuries account for over 30% of all potentially productive years of life lost (PPYLL) due to premature mortality in China [7]. The Zhejiang Health Statistics Yearbook ranks death from injury fifth among all causes of death in Zhejiang Province, accounting for 9.89% of total mortality recorded in The Zhejiang Chronic Disease Surveillance Information and Management System in 2013. Furthermore, injury is the top cause of death among those aged 0–18 years. Deaths from injury not only seriously threaten public health but also lead to a significant social and economic burden [8].

During the 12th Five-Year Plan of China [9], the Chinese government has increased the amount of attention paid to the improvement of residents’ health and has set a goal of increasing life expectancy by one year. It will take effective health policies to achieve this goal. Injuries are one of the leading causes of death in Zhejiang Province, and this significantly affects life expectancy. The government can adopt effective measures of prevention and intervention to reduce injury-related deaths.

Limited research on the impact of injury-related deaths on life expectancy exists at the provincial level [10–16]. The purpose of our study is to examine the impact of injury-related deaths on the life expectancy of residents in Zhejiang Province using death and population surveillance data. Based on this study, we will carry out resident health surveys to calculate healthy life expectancy (HALE) and its influencing factors.

## Methods

### Data collection

Mortality data was collected from the Zhejiang Chronic Disease Surveillance Information and Management System, which was published by the Zhejiang Provincial Centre for Disease Control and Prevention (CDC) [17]. Zhejiang Province is comprised of 90 districts. Taking into account the social system, economics, population, education, health and other indicators, the data was organized hierarchically using correlation analysis, factor analysis and k-means clustering. Random cluster sampling methods were used to extract a variety of combinations of samples, using a comparison of sample statistics and the overall parameters to determine the sample size. The results showed that 30 representative districts (12 in urban areas and 18 in rural areas) were selected as surveillance regions in which the prospective population-based cause of death surveillance system that was established, covered 16.6 million residents (36.9% of the population in Zhejiang Province).

Death information is collected as follows: when a patient dies in the hospital, a doctor will write a ‘medical certificate of death’ based on the patient’s cause of death within seven days. The proportion of in-hospital deaths in our study was 47.68%. When someone dies outside of the hospital, a community doctor regularly reports the information [18]. According to the deceased’s family and other residents,the general practitioners in township hospitals or community health service centres would diagnose the cause of death and fill in the ‘medical certificate of death’. The proportion of these deaths in our study was 52.32%. Causes of death were coded from V01 to Y89 based on the International Classification of Diseases, the 10th edition. Major causes of death included injury (ICD-10 codes V01-Y89), road traffic injuries (RTIs) (V01-V04, V06, V09-V80, V87, V89, V99), Falls (ICD-10 codes W00-W19), drowning (W65-W74), poisoning (X40-X49), fire (X00-X09), suicide (X60-X84), accidental suffocation (W75-W77, W81-W84), assault (X85-Y09), and undetermined intent (Y10-Y34) [6].

In practice, under-reporting is likely to occur in the Zhejiang Chronic Disease Surveillance Information and Management System, so the true mortality rates were under-estimated [19]. This study used the amount of under-reporting to adjust the crude mortality rates. The derived formula was the adjusted mortality rate = crude mortality rate / (1 - rate of underreporting). The manuscript used mortality rates with under-reporting estimated at 4.93%. . In 2013, for all deaths, the proportion of all causes of death was ill-defined at 3.30%. The proportion of deaths due to unspecified injuries was 0.69%.

### Calculation of life expectancy

We used adjusted mortality rates to calculate life expectancy using the standard life table technique. Data were analysed using EXCEL 2010. Our study used the data of injury-related deaths from which we subtracted injury-related deaths from all-cause deaths to calculate cause-removed life expectancy using the accepted procedure. [20, 21]. The key to calculating cause-eliminated life expectancy is to calculate the survival probability of every age group after removing a cause of death. We also estimated the impact of the different sub-populations and the different categories of injury on life expectancy.

## Results

### Life expectancy of the Zhejiang Province population

The life expectancy of residents of Zhejiang Province was 77.83 years in 2013 (75.72 years for males, 80.30 years for females). Estimated life expectancy for urban and rural residents was 79.20 years and 77.09 years, respectively (Table 1).

**Table 1:** The life expectancy at birth of Zhejiang Province residents in 2013 (years)

Districts	Male	Female	Total
Urban	77.18	81.52	79.20
Rural	74.94	79.62	77.09
Total	75.72	80.30	77.83

### Increases in life expectancy after removing injury-related deaths in Zhejiang Province residents in 2013

**Fig. 1:**
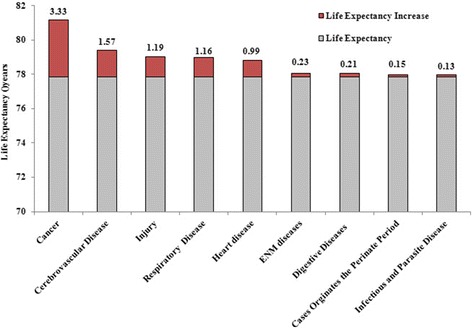
After removing major causes of death, the life expectancy of Zhejiang residents in 2013

In 2013, the mortality from injury in Zhejiang Province was 60.83 per 10^4^, and the mortality from injury in urban and in rural areas was 51.70 per 10^4^ and 65.93 per 10^4^, respectively. Females had a lower mortality rate than males (52.08 per 10^4^ vs 69.39 per 10^4^). The life expectancy of Zhejiang Province residents was 79.02 years after adjusting for injury-related mortality. The increase in life expectancy after this adjustment was 1.19 years, showing that injuries represent the third most frequent cause of death after cancers (3.33 years) and cardiovascular diseases (1.57) (Fig. 1). The life expectancy for females and males was 81.31 and 77.06 years after removing injury-related deaths, respectively, and the increase of life expectancy for females and males was 1.02 years and 1.34 years, respectively.

**Fig. 2:**
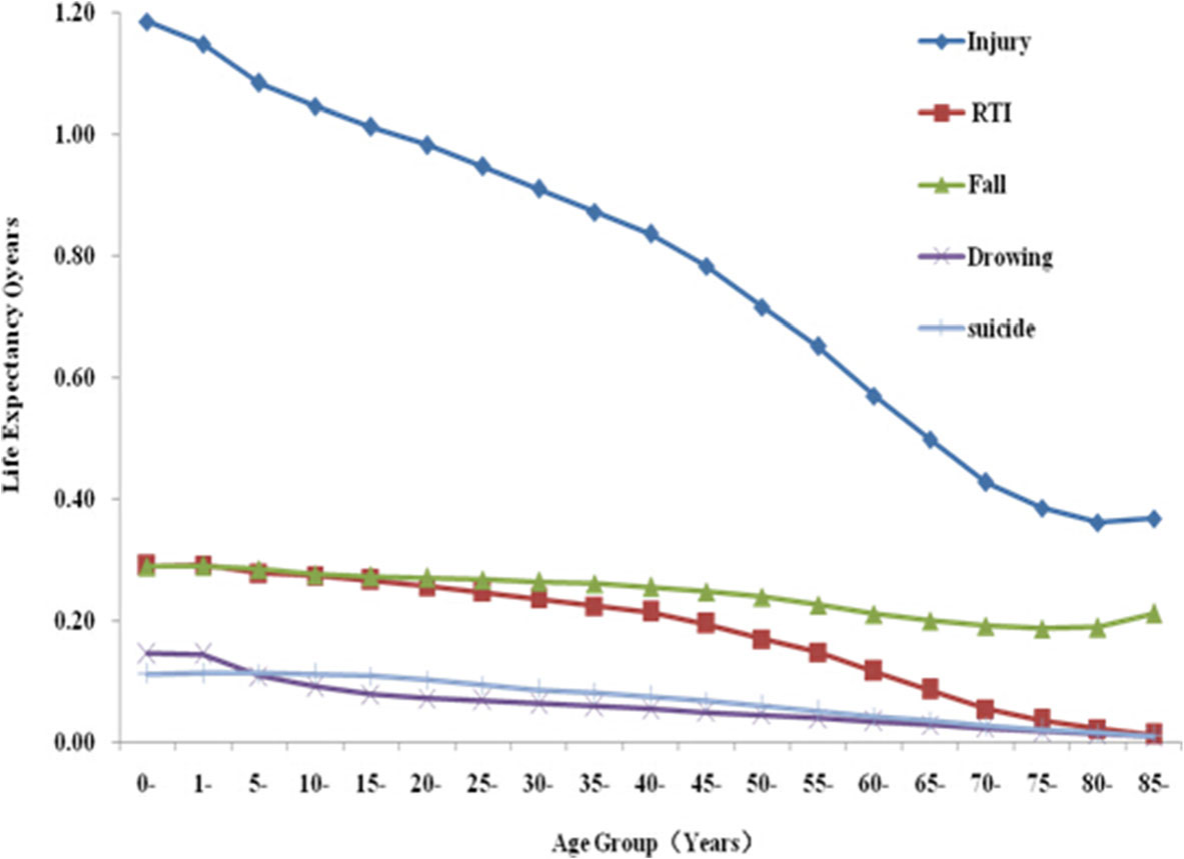
Increases in life expectancy by age after removing different categories of injury (2013)

After removing injury-related deaths, the life expectancy was 78.37 years for rural residents and 80.23 years for urban residents; the increase in life expectancy was 1.28 and 1.03 years, respectively. Furthermore, after removing injury-related deaths, the gains in life expectancy fell gradually as age increased. This study showed that deaths due to injuries had an impact on life expectancy in all age groups (Table 2, Fig. 2).

**Table 2:** After removing injury-related deaths, the increases in life expectancy for different age groups in Zhejiang Province residents in 2013

Age (years)	Life Expectancy (years)	Injury-removed Life Expectancy Increase (years)	Increase in Life Expectancy (Years)							
			Injury	Road Traffic Injury (RTI)	Fall	Drowning	Poisoning	Fire	Suicide	Accidental Suffocation
0~	77.83	79.02	1.19	0.29	0.29	0.15	0.04	0.01	0.11	0.03
1~	77.35	78.50	1.15	0.29	0.29	0.14	0.04	0.01	0.11	0.01
5~	73.48	74.57	1.09	0.28	0.28	0.11	0.03	0.01	0.11	0.01
10~	68.55	69.60	1.05	0.27	0.28	0.09	0.03	0.01	0.11	0.00
15~	63.62	64.63	1.01	0.27	0.27	0.08	0.03	0.01	0.11	0.00
20~	58.68	59.66	0.98	0.26	0.27	0.07	0.03	0.01	0.10	0.00
25~	53.74	54.69	0.95	0.25	0.27	0.07	0.03	0.01	0.09	0.00
30~	48.82	49.73	0.91	0.24	0.26	0.06	0.03	0.01	0.09	0.00
35~	43.92	44.79	0.87	0.22	0.26	0.06	0.02	0.01	0.08	0.00
40~	39.04	39.87	0.83	0.21	0.26	0.05	0.02	0.01	0.07	0.00
45~	34.24	35.03	0.79	0.19	0.25	0.05	0.02	0.01	0.07	0.00
50~	29.59	30.31	0.72	0.17	0.24	0.04	0.02	0.01	0.06	0.00
55~	25.06	25.72	0.66	0.15	0.23	0.04	0.01	0.01	0.05	0.00
60~	20.76	21.33	0.57	0.12	0.21	0.03	0.01	0.01	0.04	0.00
65~	16.66	17.16	0.50	0.09	0.20	0.03	0.01	0.01	0.03	0.00
70~	13.00	13.43	0.43	0.05	0.19	0.02	0.01	0.01	0.03	0.00
75~	9.36	9.75	0.39	0.04	0.19	0.02	0.00	0.01	0.02	0.00
80~	6.34	6.70	0.36	0.02	0.19	0.01	0.00	0.01	0.02	0.00
85~	4.06	4.43	0.37	0.01	0.21	0.01	0.00	0.00	0.01	0.00

### Impacts of different categories of injury on life expectancy

Our study showed an increase in life expectancy of 0.29 years after removing RTIs and falls, followed by drowning (0.15 years), suicide (0.11 years) and poisoning (0.04 years). The results after stratification for sex and region showed that the gains in life expectancy were 0.21 for females, 0.36 for males, 0.31 for rural residents and 0.26 for urban residents after removing RTIs (Tables 3 and 4).

**Table 3:** After removing injury-related deaths, the increases in life expectancy at birth by gender in 2013

Rank	All		Male		Female	
	Injury-related Type	Increase in Life Expectancy	Injury Type	Increase in Life Expectancy	Injury Type	Increase in Life Expectancy
1	RTI	0.29	RTI	0.36	RTI	0.21
2	Fall	0.29	Fall	0.30	Fall	0.28
3	Drowning	0.15	Drowning	0.17	Drowning	0.11
4	suicide	0.11	suicide	0.12	suicide	0.10
5	poisoning	0.04	poisoning	0.05	poisoning	0.02
6	Accidental suffocation	0.03	Accidental suffocation	0.03	Accidental suffocation	0.03
7	Fire	0.01	Fire	0.01	Fire	0.01

**Table 4:** After eliminating injury-related deaths, the increases in life expectancy at birth by region in 2013

Rank	Urban Areas		Rural Areas	
	Injury Type	Increase in Life Expectancy	Injury Type	Increase in Life Expectancy
1	RTI	0.26	RTI	0.31
2	Fall	0.28	Fall	0.30
3	suicide	0.09	suicide	0.12
4	Drowning	0.09	Drowning	0.17
5	poisoning	0.02	poisoning	0.04
6	Accidental suffocation	0.03	Accidental suffocation	0.03
7	Fire	0.01	Fire	0.01

In addition, after removing deaths due to different categories of injury, including RTIs, falls, drowning, suicide and poisoning, the gains in life expectancy were lower for females than for males. Residents from rural regions had a higher life expectancy than did residents from urban regions. However, for residents over age 65, removing deaths attributed to RTIs, falling, drowning and suicides had little impact on life expectancy. We found that removing deaths attributed to drowning had a greater impact than removing deaths attributed to falls for residents under 5 years old and those over 80 years old (Table 2, Fig. 2).

## Discussion

Life expectancy is widely used to evaluate the status of residents’ health and the burden caused by disease [22, 23]. Meanwhile, few studies have focused on the impact of removing causes of death on life expectancy in China [1, 8, 18, 24–27], especially at the national or provincial level. Injury-related death is a significant public health issue in our country. However, there have been no population-based studies that have examined the impact of deaths from injuries on life expectancy in Zhejiang Province. This research analysed the impact of injury-related deaths on life expectancy stratified by sex, age and region in Zhejiang Province. Our results indicate that injury-related deaths have a significant impact on life expectancy followed by cancer and circulatory system diseases. This information could explain the impact of disease types on life expectancy and is useful for prioritizing health-related projects for implementation by policy makers.

The results of our research showed that injury was the third largest cause of death affecting life expectancy in Zhejiang Province in 2013. The life expectancy for Zhejiang Province residents increased 1.19 years after removing injury-related deaths. Males (1.34 years) had a greater loss of life expectancy caused by injury than females (1.02 years). Our results are higher than those for most other studies in females. Wang Yuan et al. used data on injury deaths in 2010, and their research showed that the increase in life expectancy was 0.79 years for females after eliminating injury-related deaths [8]. Other research found that life expectancy increased for Italian woman after removing all categories of injury by 0.40 years [28]. Manuel et al. showed that the increase in life expectancy of women was 0.5 years after removing categories of injury in Canada [29]. Another analysis of the data obtained from The Shanghai Vital Statistics System showed that life expectancy increased by 0.56 years for females in the Yangpu district in Shanghai after removing all categories of injuries [30]. By comparison, it suggested that the loss of life expectancy caused by injury was greater in females from the Zhejiang Province than in those from other regions. We also found that the impact of injury-related mortality on life expectancy was slightly greater in males than in females. This might be because males are exposed to relatively higher risks than females [31, 32]. For example, men have more opportunities to drive and to be engaged in heavy manual work requiring high strength and are prone to more alcohol consumption. These results suggest that we should carry out health education and take some effective interventions for men.

The impact of injury-related mortality on life expectancy differs between rural and urban residents. The decrease of life expectancy in 2013 was greater in rural residents than in urban residents (1.28 vs. 1.03 years) because of injury-related deaths. We found a lower awareness of the prevalence of injury risk factors. In addition, timely treatment in rural areas was not as prevalent as in urban areas [33]. Rural residents are in the majority, so their health status has a major impact on life expectancy in Zhejiang Province. This suggests there is a need to pay more attention to preventing and controlling deaths from injury in rural regions.

Our study suggests the highest increase in life expectancy occurs when removing deaths attributed to RTIs and falls from the different categories of injuries. These results are consistent with those of other studies. Wang Yuan et al. also showed that removing RTIs resulted in the highest increase life expectancy of all the categories of causes of injury deaths [8]. Moreover, our research found that with increasing age, the loss of life years attributed to different categories of injury-related deaths gradually decreases, indicating that injury-related deaths mainly affect young residents.

In our study, RTIs had the highest impact on life expectancy in most age groups. However, drowning and falling had the greatest effect on life expectancy for children and older residents, respectively. This study provides further evidence that relevant departments should develop injury prevention strategies. The programmes should prioritize specific health care projects and funding to increase life expectancy and promote health. The Chinese government has paid more attention to the improvement of the health of residents. The government achieved the goal of increasing life expectancy by one year during the 12th Five-Year Plan of China [9]. We shall gladly make every effort towards realizing this goal. We should develop reasonable health policies to resolve the major issues. This study suggests that we should improve injury prevention programmes to reduce injury-related deaths in older residents and in children less than 5 years old. We should reduce RTIs, falling, suicide and drowning to improve life expectancy. To prevent RTIs, it is necessary to promote child safety-seat use and develop legislation to strengthen the enforcement of laws against drunk driving and not wearing seatbelts. To prevent falls, organizing the elderly to take part in the Tai Chi to improve the balance and muscle strength of is necessary. Meanwhile, it is also necessary to improve their living conditions and reduce risk factors in their environment, such as increasing the use of bathmats.

The study has several limitations. First, this study used under-reported mortality rates to adjust crude mortality rates. Our team carried out the underreporting survey of 30 representative districts of death surveillance to determine the level of under-reporting of mortality rates in 2013. However, the adjusted data may still be under-estimated. Second, a small proportion of subjects who died outside of a hospital had the wrong cause of death listed. Third, we did not explain the impact of injury risks, and as the districts selected were in Zhejiang Province, our findings are not generalizable to the broader Chinese population. Therefore,in the future, we will use disabled adjusted life years (DALY) and health adjusted life expectancy (HALE) to evaluate the disease burden.

## Conclusions

Injury-related deaths have a major impact on life expectancy in Zhejiang Province. After reducing injury-related deaths, the life expectancy will likely be increased, with special attention on reducing road traffic injuries and taking preventative actions with children under five years old and the elderly aged over 65 through measures such as public education and modification of behaviours.

## Abbreviation

RTI: Road Traffic Injury

## References

[1] Liu P, Li C, Wang Y (2014). The impact of the major causes of death on life expectancy in China: a 60-year longitudinal study. BMC Public Health.

[2] Forster DP (1992). Income distribution and life expectancy. BMJ.

[3] Sede PI, Ohemeng W (2015). Socio-economic determinants of life expectancy in Nigeria (1980 - 2011). Health Econ Rev.

[4] Tian K (2011). The average life expectancy change of Chinese population and its influence on life insurance. Chin Insur.

[5] World Health Organization (2008). Violence, injuries and disability biennial report, 2006–2007.

[6] Yin ZX, Wu J, Luo JS (2015). Burden and trend analysis of injury mortality in China among children aged 0–14 years from 2004 to 2011. BMJ Open.

[7] Wang SY, Li YH, Chi GB, Xiao SY, Ozanne-Smith J, Stevenson M (2008). Injuryrelated fatalities in China: an under-recognised public-health problem. Lancet.

[8] Wang Y, Ji CR, Zhou MG (2014). The nationwide impact of injury-related deaths on average life expectancy in China. Biomed Environ Sci.

[9] The Twelfth Five-year Plan for National Economic and Social Development, the People’s Republic of China. [http://www.gov.cn/2011lh/content_1825838.htm]. Accessed on 12 May 2016.

[10] Li J, Luo C, de Klerk N (2008). Trends in infant/child mortality and life expectancy in indigenous populations in Yunnan Province, China. Aust N Z J Public Health.

[11] Fan J, Li GQ (2014). Liu J1, Wang W, et al. impact of cardiovascular disease deaths on life expectancy in Chinese population. Biomed Environ Sci.

[12] Zhao D, Liu J (2014). The burden of cardiovascular disease and its impact on life expectancy in China. Eur Heart J.

[13] Canudas-Romo V, Liu L, Zimmerman L, Ahmed S, Tsui A (2014). Potential gains in reproductive-aged life expectancy by eliminating maternal mortality: a demographic bonus of achieving MDG 5. PLoS One.

[14] Xu Y, Zhang W, Yang R (2014). Infant mortality and life expectancy in China. Med Sci Monit.

[15] Fang XH, Zimmer Z, Kaneda T (2009). Stroke and active life expectancy among older adults in Beijing. China Disabil Rehabil.

[16] Wen H, Zhang YX, Pan et al. JX Influence of major death causes on life expectancy in residents from surveillance points in Guizhou, 2012, Modern Preventive Medicine. 2014;41(7):1304–7. (In Chinese).

[17] Yu M, Zhao HJ, Rao KQ (2002). Selection of public health surveillance sample for Zhejiang Province. Chin J Health Stat.

[18] Li GQ, Fan J, Liu J (2014). Impact of cerebrovascular disease mortality on life expectancy in China. Biomed Environ Sci.

[19] Wang L, Ma LM, Zhou MG (2011). Characteristics of under-reporting of mortality surveillance from 2006 to 2008 in China. Chin J Prev Med.

[20] Zongzan N (2001). Health statistics.

[21] Chiang C (1984). The life table and its applications.

[22] Wilkinson RG (1992). Income distribution and life expectancy. BMJ.

[23] Deaton A (2003). Health, inequality, and economic development. J Econ Lit.

[24] Wang WX, Wu KX (2011). Analysis on average life and death cause-eliminated life expectancy in Qishi. Corps Med.

[25] Chen XF, Wang J, Yang ZY (2007). Analysis on injury death causes in Pengzhou from 2002 to 2006. J Occup Health Damage.

[26] Zhang Y, Wang LL (2009). Analysis on death cause-eliminated life expectancy form. Chin Prev Med.

[27] Wang HG. Study on the Influence of Main Diseases to Life expectancy of Chinese Residents. Peking Union Medical College. 2011. (In Chinese).

[28] Conti S, Farchi G, Masocco M (1999). The impact of the major causes of death on life expectancy in Italy. Int J Epidemiol.

[29] Manuel DG, Schultz SE, Kopec JA (2002). Measuring the health burden of chronic disease and injury using health adjusted life expectancy and the health utilities index. J Epidemiol Community Health.

[30] Guan XY, Li H (2012). The main cause of residents death and loss of years in shanghai Yangpu district during 2010. Pract Prev Med.

[31] Fenelon A, Chen LH, Baker SP (2016). Major causes of injury death and the life expectancy gap between the United States and other high-income countries. JAMA.

[32] Hu Y, Wu L, Yu X (2011). Analysis of injury death trends among women in Macheng City, China, 1984-2008. BMC Public Health.

[33] Wang N (2010). National Disease Surveillance System: the cause of death surveillance data sets in. Beijing: Mil Med Sci.

